# …And Breathe! Wellness, the Nation, and Holistic Reproductive Practitioners in India

**DOI:** 10.1007/s11013-026-10021-4

**Published:** 2026-09-03

**Authors:** Adhvik Shetty

**Affiliations:** Department of International Development, University of Oxford, Oxford, UK

**Keywords:** Reproductive governance, Wellness, India, The nation

## Abstract

Garbh Sanskar (GS) is an Indian holistic reproductive programme that claims roots in ancient Ayurvedic and yogic traditions while drawing upon contemporary wellness practices and international health policy. GS centres offer breathwork, guided meditation, and counselling to expectant and hopeful couples. This article draws upon interviews with GS practitioners across nine Indian cities to examine how these practitioners use wellness discourses to articulate strategies of individual and national improvement. I argue that GS practitioners seek to interweave soteriological and wellness logics to produce enriched reproductive subjects for a therapeutic vision of nation-building. This project uses wellness to assuage the anxieties of modernity, suggest civilisational exceptionalism, and articulate aspirations for the nation. Together, GS illustrates the emergence of a bio-spiritual therapeutic politics where wellness is part of reproductive governance.

## Introduction

It is 1 pm. My eyes are closed, my legs are crossed, and I am floating 2 metres in the air.

The machine suddenly shudders into motion. A lever lowers me gently to the ground. The operator smiles. She removes the crown of metal and wire connecting me to the machine. I uncross my legs, step off the machine, and return to the ground. A man with a crisp ironed shirt congratulates me.

‘You reached *nirvana* for two minutes’, he says.

The machine is one of many in a huge multi-storey building nestled in the lush, green mountains of Western India. A spiritual organisation built the complex to support its extensive catalogue of services. Its most popular offering is *Garbh Sanskar* (GS). Garbh Sanskar (GS) is a holistic reproductive programme which draws upon ancient Ayurvedic and yogic traditions while simultaneously reworking international health policy and technology. The *nirvana* contraption, for example, combines yogic meditation, ancient South Asian philosophy, and machinery. Patients meditate while sitting on the platform. The platform rises if the machine’s sensors determine the patient has relaxed and focused their mind. The programme director (Lonavala, 12/9/23) argues that this mental training improves parental mental energies, which translates into moral, virtuous future children through social and biological changes. He then points to a fountain in the courtyard as we walk out of the complex. Water gushes out of outstretched cement palms. He explains the fountain symbolises the need to give back to society. The fountain encapsulates the ethos of GS. Practitioners articulate their work as a social service: they help couples conceive the children they desire who are, in turn, envisioned to then contribute to the nation.

Practitioners then weave together wellness, reproduction, duty, and social belonging. GS imagines that these improved future citizens will economically, morally, and spiritually benefit the nation. One GS practitioner (Ahmedabad, 5/10/23) succinctly captures this vision: ‘if you would be able to instil good values in your child’ you make ‘our society better, our nation better’. Another practitioner (Lonavala, 18/10/23) similarly argues that the wellness offerings of GS are a ‘medium for social transformation’ which works by improving the ‘human being’ who then has a ‘very good impact on the entire nation’. GS practitioners envision that wellness interventions in parents can induce changes in their future children for national transformation.

GS centres offer a wide range of services. These offerings include Ayurvedic purification techniques, dietary advice, and physical instruction. Wellness practices are a key component of these services and include breathwork (*pranayama*), guided meditation, prayer, chanting, massages, and counselling. These methods aim to improve future children through therapeutic interventions across the reproductive cycle, including pre-conceptional, pregnancy, and postpartum guidance. I use the terms ‘wellness’ and ‘therapeutic’ to describe interventions which are instruments of sociality, well-being, self-making, and emotional support, including those outside of formal psychological disciplines. This definition follows Duncan’s (2018) concept of psychological globalisation, which describes how medically plural, transnational processes shape well-being and what it means to be human. The literature review expands on this work in more detail to illustrate how GS aims to institute individual and social improvement through wellness.

There is limited academic literature examining GS. However, Subramaniam (2019) briefly situates GS within the postcolonial reworking of mythology, science, and reproduction in India. This analysis positions GS as the reproductive component of a larger project of imagining an ancient yet scientific Hindu–Indian civilisation. Mohanka (2022) further examines GS as a Hindu nationalist reproductive project. She draws upon media, grey literature, and public discourses to demonstrate how proponents of GS frame the womb as a site for cultural, religious, and national regeneration. These studies argue that GS produces a public reproductive imaginary which combines understandings of ancient knowledge, Hindu identity, foetal development, and national revivalism. This article builds on this work. However, I shift attention from public GS discourses and representations to the practitioners who deliver, translate, and operationalise these claims. I examine how practitioners describe their services, understand their impact, and connect their work to foetal, family, and national development.

What does it then mean to improve the nation through wellness? This paper uses GS to examine how wellness discourses are reworked into developmental strategies. The article first situates GS within literatures of reproductive governance, postcolonial nation-making, wellness, medical pluralism, and kinship. I then develop four arguments to organise the article and illustrate how GS mobilises wellness as a technology of reproductive governance. First, GS breathwork merges soteriological and wellness logics to produce what practitioners articulate as cosmologically enriched reproductive subjects. Second, GS uses Ayurvedic and spiritual wellness practices to weave both modern scientific legitimacy and an ancient civilisational exceptionalism into this project to produce enriched reproductive bodies. Third, GS uses wellness to situate this project within the regulation of gender, kinship, and care as a mechanism for national improvement. Fourth, GS biologises these bio-spiritual, civilisational, and gendered wellness discourses through an epigenetic imaginary. These arguments collectively illustrate how GS practitioners rework wellness discourses into reproductive technologies to reconfigure the boundaries between contemporary wellness and traditional medicine, and between individual well-being and national development, to produce a bio-spiritual therapeutic politics of reproductive governance.

Wellness circulates, evolves, and becomes reworked transnationally. The link between wellness and reproduction in GS resonates with other projects which operate through analogous logics, such as in Australia, the US, and the UK (Highet & Purtell, 2012; Maternal & Child Health Bureau, 2024; National Health Service, 2018). These projects are inflected with their own politics and histories but similarly position maternal mental well-being as a determinant of foetal and national development. The discussion section of this article will explore these global comparisons in more detail and illustrate how these global discourses extend the individual mindful body to the social body (Scheper-Hughes & Lock, 1987). GS then provides a microcosm to explore this global relationship between wellness, reproduction, and national development, while localising this link by using wellness to expand reproduction beyond the biological into the realms of the moral and civilisational. GS perhaps signals the emergence of a bio-spiritual therapeutic politics whereby wellness is intimately intertwined with the governance of biological reproduction.

## Wellness, Reproduction, and the Nation

This section contextualises Garbh Sanskar as a method of reproductive governance which mobilises wellness within a political context of postcolonial nation-making. This project of reproductive governance operates through idioms of kinship and foetal subjectivity. ‘Reproductive governance’ describes how states, social movements, and other actors regulate reproduction through legal, medical, and cultural mechanisms (Morgan & Roberts, 2012). These processes often instrumentalise women’s bodies in service of the nation, and are gendered, racialised, and classed (Yuval-Davis, 1997). Reproductive governance in India is perhaps best demonstrated by the 1976 Constitution Act during the Emergency, the 21-month suspension of democracy in India. The Act led to an infamous family planning campaign legitimised in the name of economic development, women’s empowerment, and national duty. The campaign sterilised 8.1 million people in 1977 alone, often through coercion (Gupte, 2017). GS marks a shift in Indian reproductive governance. The programme aims to increase rather than decrease fertility, represents non-state reproductive interventions, and emphasises regulation through affective, therapeutic discourses instead of coercion.

The conceptual association of reproduction and wellness with an ancient Indian identity and the nation reflects links between GS practitioners and Hindu nationalism (Mohanka, 2022). Some of these clinics and practitioners are affiliates of politicised Hinduism. An influential Hindu nationalist organisation delivers GS programmes through their health wing (Banerjie, 2017; GS Practitioner, Thane, 24/01/24). A leading figure in a Hindu nationalist women’s organisation also promoted GS and claimed that ‘[b]y practising *garbh sanskar* for 1,000 days, or nine months of pregnancy and two years after childbirth, we can improve the coming generations’ (Express News Service, 2023: para. 2). The governor of an Indian state, who is a member of the ruling Hindu nationalist party in India, argued that GS would produce *deshbhakt* (patriotic) children (Press Trust of India, 2023). Furthermore, the founder of a Hindu nationalist party, Shiv Sena, supported the launch event for a key GS text (Dabholkar, 2011). Mohanka (2022: 62) accordingly described GS as a Hindu nationalist manifestation of the state-mediated ‘reproductive-temple-corporate-industrial-complex’.

Hindu nationalism is not only an electoral project. Anderson and Longkumer (2018) outlined how Hindu nationalism increasingly enters diverse domains of public life. Chacko (2019) argued that Hindu nationalism positions the individual as an entrepreneurial subject whose self-discipline strengthens the Hindu nation. Puri (2019) further elucidated how Hindu nationalism imagines an ancient but great Hindu civilisation. GS extends this logic into reproduction. GS articulates aspirations of reforming public *and* reproductive private lives, links reproductive responsibility to the development of the nation, and imagines its reproductive and therapeutic practices as an inheritance of an ancient Hindu–Indian wisdom. Furthermore, GS’s link between reproductive labour and national regeneration speaks to broader Hindu nationalist gender ideologies which position women as central to the reproduction of national culture (Bacchetta, 2004; Sarkar, 2001). GS therefore demonstrates how Hindu nationalism can operate through the micro-politics of everyday life: intimate projects of gendered discipline, family formation, well-being, and reproductive aspiration (Chakravarty, 2020).

However, GS is not always an explicitly Hindu nationalist project. For example, one centre describes themselves as an agnostic, socialist organisation (GS Practitioner, Lonavala, 12/09/23). This complicated relationship between GS and Hindu nationalism is part of a longer history of debates over the Indian nation. Khilnani (1997) argued that anti-colonial nationalism generated competing ideas of India: a secular and modernising state, a moral-spiritual nation, and a Hindu chauvinist civilisation. These competing visions for India often intersect. Even secular or pluralistic nationalisms draw upon ancient identities as a cosmology to create a better modern nation-state (Prakash, 2020). GS, as a method of national improvement, is therefore not reducible to Hindu nationalism though it often travels through Hindu nationalist idioms. GS’s significance lies less in formal political affiliation than in the way wellness and reproduction become a site for imagining Hindu-inflected national renewal.

These ideas of the nation are part of anti-colonial and postcolonial nationalisms that draw upon and rework ideas of the ancient and modern. Bhabha (2004 [1994]: 9) wrote that ‘cultures of a postcolonial *contra-modernity*’ were ‘otherwise than modernity’; they hybridised both aspirations for and critiques of modernity. Chatterjee (1993) similarly argued that anti-colonial nationalisms pursued a ‘modern’ material outer domain of science, technology, and statecraft while simultaneously postulating a ‘traditional’ spiritual inner domain of religion, family, language, and culture. Said (1985) had claimed that the ‘Occident’ discursively portrayed the ‘Orient’ as religious and spiritual to produce a self-image of the Occident as scientific and rational. Chatterjee’s analysis shows how postcolonial nationalisms themselves meld self-images of religiosity and spirituality with understanding of science and rationality. Subramaniam (2019) localised this discussion in India and described how the ‘archaic modernity’ of Hindu nationalist imaginaries combines modern scientific idioms with mythological Hindu claims to produce a vision of India as both technologically advanced and culturally ancient. GS participates in this hybridised reworking of ‘ancient’ knowledge into ‘modern’ political and developmental projects by producing a ‘traditional’ inner domain of wellness and reproduction for the revival of the modern nation.

Furthermore, the medical pluralism of the wellness offerings within GS speaks to a longer postcolonial history of epistemological hybridity and the translocal production of techno-sciences (Anderson, 2002; Gupta, 1998). For example, Mukharji (2016) demonstrated how ‘traditional’ Indic sciences braided together a multiplicity of knowledge systems to produce their version of modernity. The reinterpretation of tradition produced Indian scientific knowledges as the heritage and symbol of the postcolonial nation (Prakash, 2020). What looks like a return to tradition is often a careful, selective production of modern postcolonial nation-making. GS presents wellness therapies in reproductive programmes as both modern scientific interventions and recoveries of an ancient Indian wisdom. This selective deployment, reinvention, and co-optation of ‘tradition’ within contemporary nationalist reproductive projects builds upon older postcolonial legacies of imagining a hybridised nation which is irreducibly both modern and Indian.

GS wellness therapies then aim to intervene in reproduction for national revival. This intervention is part of a broader interaction between wellness and governance. Foucault (2006 [1973]) argued that a diffuse network of therapeutic institutions (the psy-function) exerted a normalising power over the family and the individual. Rose (1999) drew upon Foucault but argued that these therapeutic institutions did not foreclose subjectivity. They instead instilled dispositions to enable citizens to understand and regulate themselves through the promise of freedom, autonomy, and fulfilment. This insight demonstrates how GS practitioners do not govern by force: they instead aim to teach patients to transform themselves into spiritual, disciplined, and calm parents for the future child. These insights into therapeutic discourse help illustrate how GS wellness offerings aim to shape reproduction through cultivating self-discipline.

Illouz (2008: 6) had argued that ‘the therapeutic discourse transcends national boundaries and constitutes a “transnational” language of selfhood’. However, postcolonial societies can localise therapeutic discourses. Duncan (2018) outlined how Euro–American therapeutic discourses interacted with particular sociocultural, ethnic, linguistic, and medical histories as part of psy-globalisation to produce culturally rooted ways of knowing and working on the self and soul. Duncan disaggregated psy-globalisation into psychological and psychiatric globalisation. The former is most relevant to GS, and describes how psychological and New Age, alternative, and pop-psychological therapeutic systems view the self as exposed to imbalance, but capable of self-knowledge, self-responsibility, and self-transformation. She further argued that these patterns of psy-globalisation created forms of psy-sociality: group identities facilitated by practitioners and based on a ‘general desire to engage in practices of self-work, healing, and emotional cultivation’ (Illouz, 2008: 43). GS similarly braids together different histories to produce therapeutic systems which aim to instil self-transformation under the guidance of practitioners.

GS resonates with psy-globalisation, but its therapeutic idioms are also shaped by South Asian histories. For example, Gupta and Copeman (2019: 314) argued that yogic practitioners often advocated for wellness regimes to create a ‘nationalist subject’ through ‘bodily formation’. Green (2008: 285) further argued that Yogis and Sufis in India promoted breathing practices as ‘distinctly Hindu and Muslim body practices’ which created ‘a shared movement towards the indigenisation of physical culture in the face of colonial British modes of personal conditioning’. Both yoga and breathing practices speak to Alter’s (1994: 559) depiction of somatic nationalism as a ‘utopian vision of nationalistic reform that takes the body as a primary object of discipline and reform’ in India. GS builds upon these studies of somatic nationalism to demonstrate how wellness incorporates bodily practices into nation-building through reproduction.

The therapeutic systems of GS aim to enact national transformation through new forms of kinship. Franklin (1997) argued that assisted reproductive technologies (ART) created new persons and relations while embodying values of progress, kinship, and futurity. Franklin and McKinnon (2001) also claimed that these technologies expanded the conceptions of substance and biology underpinning kinship. Thompson (2005: 9) further described the processes of ‘ontological choreography’ within ART clinics. These clinics co-ordinated ‘the technical, scientific, kinship, gender, emotional, legal, political, and financial aspects’ of parenthood to create ‘new ways of making parents’ (*ibid*). GS is not a biomedical ART programme but similarly experiments with novel forms of reproductive care and kinship making to create new persons, produce potential parents, and embody ideals of national progress using the language of wellness.

GS is also situated within larger kinship systems in India. Uberoi (1996, 1998) argued that the symbol of the traditional Indian family (often a patrilineal joint family) produced a moral and spiritual signifier of national belonging. However, Donner (2008) argued that middle-class values increasingly reified nuclear family patterns coded as ‘Western’ while simultaneously expressing anxieties around the decline of the joint family and its associations with a cultural identity and intergenerational care. Sinha Roy (2023) further outlined how nation-building in India drew upon an essentialised conception of Indian family values and positioned the family as a site for the discursive formation of both tradition and modernity. This form of nationalism echoes Chatterjee’s (1993) and Subramaniam’s (2019) discussion of hybrid postcolonial subjectivities, but at the level of the family. This political potential of kinship further comes from care’s gendered role in the production and reproduction of society through relationality, reciprocity, exchange, morality, and affect (Addlakha, 2020). GS similarly reifies the family as a component and symbol of the nation, operates within a perceived affective vacuum of modernity, weaves together responsibility and the family, and aims to reproduce and revitalise society through systems of care.

The image of the foetus is at the heart of these kinship-making processes. Social worlds determine ways of relating to the foetus (Morgan, 1996). For example, Taylor (1998) argued that the prenatal paradox constructed the foetus as both a commodity and person, which in turn legitimated the instrumentalisation of women’s bodies; ‘The mind of a woman thus appears to provide one therapeutic route to the body of the fetus’ (*ibid:* 20). This logic positioned the body of the foetus as a site of improvement as both a commodity to be optimised, and a person as deserving of attention and care. Rapp (1999) further argued that prenatal diagnostic techniques forced women to judge the ‘quality’ of their foetuses under a logic of ‘normalcy’ to decide who could be part of the social community. Foetal subjectivity has further evolved with the emergence of epigenetic knowledge, which examines the interactions between the environment and genome. Epigenetic links between prenatal environments and the long-term health outcomes of the foetus constructs a burden of maternal responsibility for future generations (McMahon, 2024; Turkmendag & Liaw, 2022). GS similarly draws upon the image of the foetus but mobilises wellness to articulate new forms of foetal subjectivity, optimisation, care, and responsibility.

GS practitioners use wellness and reproduction to imagine bodily and national improvement. This form of reproductive governance draws upon postcolonial reworkings of the ancient and modern, medically plural therapeutic strategies, gender and kindship, and the reimagination of foetal subjectivities. The article builds upon these histories to theorise how wellness is an intimate component of politics, identity, and reproduction.

## Methodology

Previous studies of Garbh Sanskar have engaged with press releases, grey literature, and social media content (Mohanka, 2022; Subramaniam, 2019). This article instead draws upon fifteen semi-structured interviews with GS practitioners across nine Indian cities between August 2023 and January 2024: Bangalore, Indore, Ahmedabad, Kochi, Lonavala, Mumbai, Thane, Pune, and Nellore. These practitioners ran GS programmes which addressed both couples trying to conceive and those already pregnant. Practitioners commonly described GS as a lifecycle intervention (*sanskar* means life stage): it begins before conception, continues through pregnancy, and sometimes extends into the postpartum period.

The article analyses how GS practitioners aim to mobilise wellness in reproductive governance across this reproductive lifecycle. Patients are not included as research participants in this article; the study does not claim to measure how patients receive, internalise, negotiate, or resist these programmes. The article is instead concerned with the kinds of parents, foetuses, children, families, and social bodies that GS practitioners seek to produce. I follow Nader’s (2018 [1974]) argument that studying elites is an important part of social research. Studying ‘up’ in holistic reproductive centres illuminates the aspirational mechanisms of individual and social bodily transformation within GS.

This focus necessarily limits the scope of the argument. I do not claim that patients passively accept GS teachings, nor that practitioner aspirations straightforwardly become patient subjectivities. Patients may engage GS for reasons that are more pragmatic, medical, familial, spiritual, or affective than nationalist. They may also reinterpret, selectively adopt, or reject practitioner advice. Furthermore, patients might find GS genuinely meaningful, emotionally sustaining, and culturally resonant. I also do not assess the biomedical efficacy of claims within GS. The article only aims to explore the cosmologies of practitioners to understand *how* they use and imagine therapeutic vocabularies within this project.

I used criterion sampling to identify practitioners who actively offer and promote GS services (Patton, 2002; Small, 2009). Criterion sampling identified information-rich cases and practitioners who were able to articulate the aims of GS. The sample reflects the geographic and ideological diversity of GS. Practitioners were based across India and ranged from independent Ayurvedic consultants to practitioners affiliated with Hindu nationalist organisations. Participants are identified by city and interview date to preserve confidentiality while situating their practice geographically. I gathered written or oral informed consent from all participants prior to data collection and the project received research ethics approval from my research institution [Institutional review board identity and research approval code redacted for anonymous peer review].

GS is embedded in a wider reproductive field in which pregnancy, care, and foetal development are strongly gendered. Two-thirds of the interviewed practitioners were women. I did not systematically collect caste or ethnicity data, and I do not make claims about the caste or ethnic composition of GS practitioners. However, the sampled practitioners primarily resided in Western India, the heartlands of the ruling Hindu nationalist party.

GS practitioners also have diverse professional backgrounds. I spoke to biomedical doctors, Ayurvedic *vaidyas* (physicians), and spiritual gurus. These practitioners operate in distinct centres. The waiting room of one centre feels like a spa. Gentle Hindustani classical music plays softly in the background. A diffuser fills the room with the fragrance of perfumed oils. The practitioner (Thane, 15/12/23) points to the luxurious bottles which line the walls. She explains that she has recently launched a new Ayurvedic cosmetics range. Another centre, by contrast, is more rustic. The centre is lively and popular, and the waiting room is filled with sounds from the quiet chatter of patients, the traffic outside, and the receptionist calling out patient numbers. A staff member brings out a *sambrani* burner instead of a diffuser. The burner is filled with hot coal and resin to expel negative energy and repel insects. Smoke fills the room and the waiting patients cough. The walls are lined, not with cosmetics, but with localised South Indian depictions of Sanskritic Gods. GS centres do not just vary according to atmosphere. Some are large, sprawling compounds. These compounds sometimes spread across multiple ‘campuses’ in a city. Other centres, however, are small one-room clinics housed in shopping centres. Even the physicality of the centres is not guaranteed. Many centres offer in-person appointments, but some only provide online services. GS practitioners and centres use the same term to describe their offerings, but they operate as a plural field of reproductive wellness and do not take one standardised form.

## A Breath of Fresh Air: Pranayama and Bio-Spiritual Politics

Disparate Garbh Sanskar clinics draw upon breathing as a form of reproductive governance. These practitioners argue that instilling yogic regimes of breathing (*pranayama*) before and during pregnancy can act as a therapeutic tool to instil mental wellness in parents and developmental changes in their future children. Pranayama translates ‘as something like “control of the life force”’ and serves a ‘principal purpose of purification (*tapas*) for both therapeutic and soteriological purposes’ (Miller, 2023: 119). Pranayama then promises both mental relaxation and salvation. GS practitioners fold pranayama into their programmes to transform breathing into a reproductive tool that they understand as simultaneously therapeutic and cosmological.

For example, one GS practitioner’s (Indore, 20/8/23) pranayama programme combines breathing with guided meditation, which solidifies the link between pranayama and therapeutic interventions. His assistant asks me to attend one of his online classes before we formally speak. I join the Zoom class. The practitioner is the sole image. He tells patients to close their eyes and observe their own breathing. He asks patients to keep their spine straight during deep breathing so that their ‘energy can circulate’. He explains that the left nostril is the *Chandra* (moon) nostril, which is *tundi* (cold). The right nostril, by contrast, is the *Surya* (sun) nostril, which is *garam* (hot). He asks participants to close their right nostril, breathe from their left nostril, then expel negative energy through their right nostril. This cosmology of the body infuses therapeutic interventions with a celestial, spiritual quality to purify the body. This pedagogical therapeutic logic also echoes Rose’s (1999) work on the governance of subjectivities. However, the practitioner does not just seek to shape dispositions*,* but biological-spiritual-cosmological states. GS imagines pranayama as a form of cosmological reproductive purification and self-optimisation.

The practitioner (Indore, 20/8/23) further argues that deep breathing helps the lungs to ‘expand properly’ and ‘activate[s] your parasympathetic nervous system to put your body in *shanti* [peace] mode’. This argument combines yogic breathing practices with biomedical systems of the body to link therapeutic interventions to both the spiritual and biological body. The desired activation of the *shanti*-parasympathetic nervous system aims to create reproductive subjects who are biologically and spiritually optimised in this syncretic project of bio-spiritual politics. GS values these regimes of bio-spiritual wellness because of their neurological potential: the practitioner (Indore, 20/8/23) promises that the relaxation from pranayama will ‘develop your babies’ brains a lot’. The therapeutic and soteriological regimes of breathing therefore aim to enact a joint biological, spiritual, and neurological scheme of comprehensive improvement. The result, in GS imaginaries, is a form of human development that extends beyond the biological and cognitive into the cosmological: a population that has not only been improved physically and neurologically but has acquired a metaphysical cultural comparative advantage through this therapeutic energetic formation. This imaginary transforms pranayama into a bio-spiritual form of reproductive governance.

The use of breathing in national self-making has historical precedents in South Asia, as outlined in the literature review. GS builds upon this tradition of somatic nationalism to imagine bio-spiritually optimised reproductive bodies and recruit these bodies into aspirations for national development. The practitioner who corrects his patients’ breathing on Zoom enrols their bodies, respiratory tracts, nervous systems, and metaphysical energies into a vision for national biological and cosmological development: the practitioner (Indore, 22/08/23) hopes that the classes will ‘give birth to a billion babies’ for the nation. He positions breathing practices as traditionally Indian interventions to improve future children, which combines the spiritual and biological into a project of therapeutic nation-making. These processes then demonstrate how Chatterjee’s (1993) and Subramaniam’s (2019) hybrid forms of postcolonial nation-making can mobilise the registers of wellness and reproduction. GS reconfigures pranayama into a wellness intervention for reproductive governance to imagine the bio-spiritual improvement the nation.

## Wellness, Modernity, and the Ancient

Alongside yogic breathing techniques, Garbh Sanskar centres use Ayurvedic therapies and spiritual wellness practices for reproductive governance. Ayurvedic therapies demonstrate the role of care within reproductive governance, while spiritual practices elucidate how GS uses wellness to claim both modern scientific legitimacy and suggest ancient civilisational exceptionalism. Both forms of wellness interventions profess to soothe the mind to in turn improve reproduction.

For example, GS clinics often have a wooden bench with a pot suspended at one end. The patient lies on the wooden bench while medicated oil, water, or milk in the pot drips onto their forehead from the overhead pot. This Ayurvedic therapy is called *shirodhara,* which practitioners argue can help calm patients. Similarly, clinics also deliver *marma* therapies. Marma is an Ayurvedic massage given to various pressure points around the body, which practitioners argue can help patients relax and open their bodily energy channels. Practitioners provide shirodhara and marma therapies to ‘heal [patients], feel them relaxed, you know, make them comfortable, to go for a copulation’ (GS Practitioner, Bangalore, 12/10/23). Practitioners then position Ayurvedic therapies as a form of care to facilitate reproduction and cosmologically enrich bodies. Robbins (2013: 457-458) once called for ‘an anthropology of the good’ that documents ‘how people living in different societies strive to create the good in their lives’. A focus on the good helps reveal how GS aims to bring well-being into the lives of its patients. GS is not an unfeeling, technocratic programme of reproductive augmentation. GS’s vision for bodily and national improvement aims to alleviate bodily and mental suffering by incorporating Ayurvedic techniques into wellness offerings even as this project simultaneously enacts regulatory processes to encourage reproduction and facilitate bio-spiritual therapeutic improvement. Reproductive governance in GS operates through comfort rather than coercion.

GS practitioners situate this goal to alleviate suffering within global discourses around mental well-being. They position GS’s spiritual practices of prayer, mantra recitation, and meditation as mental well-being interventions. For example, one practitioner (5/10/23) in Ahmedabad explains that ‘Garbh Sanskar is all about mental and emotional well-being’, invoking a policy landscape in which mental well-being has become ‘a standard currency in economic and political models of welfare and development’ (Jiménez, 2008: 2). GS practitioners deploy these international frameworks strategically. The Ahmedabad practitioner first validates his GS programme by aligning it with international health policy and its growing attention to mental well-being during pregnancy. He then inverts this relationship of legitimacy: he argues that ‘modern science’ has only just ‘started focusing on mental well-being’ during pregnancy, whereas ‘our ancient science [of Garbh Sanskar] was far more advanced’. This statement is not a rejection of modernity but a reversal of an epistemic hierarchy: the practitioner accepts biomedicine and international health policy as authoritative, but only insofar as it belatedly rediscovers a forgotten Indian wisdom of spiritual wellness. This temporal reversal is common across practitioners and positions GS simultaneously as modern (aligned with cutting-edge health policy) and ancient (prior to and superior to that policy). The integration of mental well-being discourses within GS resonates with Mukharji’s (2016) concept of braiding within Indian medical pluralism: GS practitioners can draw upon different epistemic traditions (international health policy and South Asian philosophy) to construct a cosmology of an ancient yet scientific Indian national identity using wellness and reproduction. This braiding of epistemic traditions uses wellness to claim a global currency and ancient Indian exceptionalism simultaneously for its nationalist project of reproductive governance.

However, tying GS to the ethos of modernity does not imply an unfraught relationship between the ancient and the new. A practitioner (Bangalore, 12/10/23) from another centre argues that modern lifestyles cause infertility ‘because of the increased stress and other pressure; we are not able to keep up’. She opines that even when couples can conceive, these stress levels mean that there are often ‘defect[s] in the baby formation’. Other practitioners similarly claim that spiritual wellness is important because stress from modernity ‘affects the development of the child’ (GS Practitioner, Mumbai, 20/08/23). They argue that GS wellness interventions, such as meditation, can instead ensure that parents live ‘a positive, joyful, and virtuous lifestyle’, which ‘would help in the baby’s brain development’ (GS Practitioner, Ahmedabad, 5/10/23). GS then positions wellness as a bio-spiritual method of human capital development for the future child. Lasch (1979: xv) argued that the infusion of wellness discourses in modernity led to ‘the pursuit of happiness to the dead end of a narcissistic preoccupation with the self’. Wellness discourses in GS instead *instrumentalise* the self with the aim to counteract the excesses of modernity through traditional wellness practices for the good of the future child and its potential cognitive contribution to society. The cultivation of individual wellness becomes a bio-spiritual therapeutic means to imagine improved national reproduction and revitalisation.

## Gender, Kinship, and Care in Reproductive Governance

Garbh Sanskar practitioners link the threats to wellness in modernity, and the need for bio-spiritual therapeutic interventions, with changes in kinship patterns. GS then mobilises wellness to articulate social aspirations for the moral and civilisational character of India using the idioms of kinship. For example, centres often voice anxieties that family patterns in India increasingly eschew the archetype of an extended family living in the same household for a nuclear family associated with ‘Western’ modernity; centres argue that ‘joint families are gone’ (GS Practitioner, Thane, 15/12/23). One practitioner (Bangalore, 12/10/23) claims that this nuclearisation means that ‘women of this era’ have fewer sources of emotional support. She explains that she can provide ‘motherly guidance’ and spiritual advice to reduce anxieties surrounding motherhood. She suggests that this guidance can fill the social gap left by anxieties around the retreat of the extended family in modernity. GS centres then authorise bio-spiritual therapeutic interventions as normalising interventions which aim to protect the ‘traditional’ realm of reproduction, family, and the nation from the encroachment of modernity and the westernisation of kinship patterns. GS links wellness to changing kinship patterns to articulate anxieties over what the moral and cultural character the nation ought to be.

This argument is gendered. Young (2003: 2) outlined ‘the gendered logic of the masculine protector’. This logic positioned subjects into a subordinate position of submission and dependence. GS centres similarly draw upon an idiom of protection. However, the centres use a different gendered logic of protection: care. Care is a gendered ethic to navigate relationships through compassion and empathy (Gilligan, 1982). GS practitioners use the language of care to figuratively take the place of older female members within the extended family. As outlined earlier, most interviewed GS practitioners are women. The use of the term ‘motherly guidance’ invokes metaphors of feminised kinship and care, whereby practitioners position their patients as figurative daughters within the extended household. Other practitioners (Kochi, 30/10/23; Nellore, 24/01/24) refer to their patients as ‘girls’ or act as older maternal mediating figures in the family by persuading husbands to join sessions. This language creates a softer relationship of obedience than paternalism, but a relationship of obedience nonetheless; patients should listen to the wisdom of the GS practitioner as a child might listen to her mother. Care has a politics (Puig de la Bellacasa, 2017). This authority for wellness in reproduction further draws upon the affective and symbolic value of the joint family in Indian nationalist discourses (Uberoi, 1998). Practitioners did not present themselves only as medicalised, technical experts, but as maternal familial figures who could mobilise gendered therapeutic strategies to guide women through the intimacies of reproduction. GS genders and incorporates wellness into reproductive governance to recreate the idealised values of the nation through the figurative restoration of traditional kinship patterns.

GS also uses wellness, kinship, and reproductive governance to advance reformist visions for the nation. For example, one practitioner claims that ‘the happiness quotient of the women was higher’ after her GS interventions of chanting, meditation, prayer, and mindfulness practices (GS Practitioner, Thane, 24/01/24). She emphasised that this finding was especially important because she implemented the programme in a rural North Indian village where there were ‘many restrictions for the women’. She argued that these bio-spiritual therapeutic interventions encouraged women to prioritise their own well-being. A fertility and antenatal programme with associations to religiously conservative movements might be expected to weight foetal well-being over those of women, but practitioners also describe the benefits of the programme in terms of the contentment it can bring women through its mental and spiritual components. The discussion of the restrictions facing women in the village is also an implicit criticism of tradition given the centrality of the village in the history of Indian nationalist thought (Khilnani, 1997). Some GS practitioners then use wellness in reproductive governance to critique the institutions of the family and nation, even when delivering services which ostensibly speak to conservative traditions, and once more use wellness to fold care into regimes of reproductive governance.

These therapies often target women. However, Garbh Sanskar also uses wellness to recruit men into reproductive governance. For example, one practitioner explains that men cannot escape their reproductive responsibilities for wellness. She argues that ‘the man usually is king, like, he has to take care of everyone who is under him, right?’ (GS Practitioner, Bangalore, 12/10/23). However, she stresses that economic changes have challenged the feasibility of achieving this model of masculinity. She claims that the societal pressure to fulfil the male breadwinner archetype, and the shame when this ideal type cannot be realised, creates a level of mental distress which affects the quantity and quality of sperm production. She suggests that men should engage in meditation and other bio-spiritual wellness practices to improve their reproductive processes. The practitioner then locates male reproductive and social difficulties in the pressures of modern life, rather than as individual moral failure. This externalisation of stress incorporates men into reproductive governance by offering them a comforting and therapeutic narrative whereby reproductive and social challenges become a symptom of modernity. GS then uses wellness as a therapeutic means of reproductive governance to manage threats to masculinities conservatively, regulate potential paternal as well as maternal bodies, and imagine a bio-spiritual method to optimise sperm production for reproduction.

This programmatic focus on masculinities again aligns GS with contemporary debates in international health policy. Health policy now interrogates the role of gender norms and their implications, especially in relation to mental health and sexual and reproductive health and rights (SRHR). For example, *The Lancet* published a commentary on the intersection of masculinities, social norms, and psychosocial health (Ragonese & Barker, 2019). Furthermore, several UN agencies and the WHO jointly authored a report on addressing masculinities to achieve gender-transformative SRHR outcomes (Ruane-McAteer et al., 2019). GS captures these currents of international policy discourses using wellness. The reconfiguration of these discourses helps GS legitimise its reproductive governance of masculinities, male reproductive processes, and the family for its national aspirations.

GS also localises global discourses around masculinities. One practitioner (Bangalore, 12/10/23) speculates that economic anxieties might cause men to consume greater levels of alcohol and tobacco, which, in turn, might affect the ‘proper timing to perform sexual activity’. This link reflects the social lives of alcohol and tobacco as material objects in India. Gandhian independence movements associated drunkenness among men as a symptom of moral corruption and Western colonialism: both the East India Company and the British Raj raised significant revenues through the production and taxation of alcohol (Schrad, 2021). Simultaneously, British temperance movements found large audiences in colonial India (Bhattacharya, 2017; Carroll, 1976). Colonial and anti-colonial prohibition rhetoric endures today; several ‘dry’ states in India still restrict the sale of alcohol. The practitioner’s anxiety around alcohol consumption from the crisis of masculinity redeploys this moral panic into a national moral-physiological problem which affects the materiality of sexual reproduction. GS then uses wellness discourses within reproductive governance to regulate the moral conduct of the male body and manage anxieties around the morality of the nation.

This framing can itself act as a form of reproductive governance to obscure the questions of power which structure reproductive life: men’s authority over household decisions, control over women’s mobility and healthcare, and expectations of women’s obedience. The discourse that ‘the man is usually king’ leaves intrahousehold gendered hierarchies unchallenged. Furthermore, this rhetoric invites men into reproductive life as emotionally responsible fathers but does not displace the larger regulation of women’s bodies, emotions, and mental states within reproductive governance. However, other practitioners do take more gender-transformative approaches to reproduction. One practitioner (Lonavala, 18/10/23) explicitly aims to undo the perception amongst men that ‘once conception has occurred, their job is over’. He argues that these men ‘need to understand that the job has just begun’, and that they should ‘play an active role’ in creating an ‘environment in the family’ which is ‘cheerful’ and ‘peaceful’; they should cultivate wellness within their families. This stance reflects a more critical stance towards masculinities than other GS centres. It aims to redress inequalities in the division of care and reproductive labour during pregnancy, and the emphasis on creating a ‘peaceful’ home references concerns around intrahousehold gendered dynamics. This stance mobilises wellness in reproductive governance to suggest a reformation of gender roles for family and national development. GS centres then heterogeneously deploy wellness within reproductive governance to articulate visions for national change through the regulation of gender and kinship.

GS deploys wellness in reproductive governance to manage the anxieties of modernity, provide reproductive care, regulate maternal and paternal bodies, rework policy discourses to claim international legitimacy, and manage gendered moral conduct. These mobilisations of wellness collectively act as an entry point to regulate gender and kinship to in turn articulate aspirations for the moral and cultural character of the nation.

## Wellness and Molecular Imaginaries: Mental Purification and Epigenetics

Garbh Sanskar imagines that these wellness can biologise these aspirations through the bio-spiritual language of mental purification and epigenetics. For example, one practitioner (Lonavala, 18/10/23) argues that GS wellness therapies such as meditation and chanting can lead to mental purification, which happens at a ‘very subtle level’ and can improve the quality of children through ‘the science of epigenetics’. Epigenetics refers to changes in genetic expression without changes to DNA structure. Epigenetics has attracted widespread popular and scientific attention for its suggestion that environmental conditions during critical developmental periods can have lasting biological effects (Murgatroyd & Spengler, 2011). GS translates this framework into a platform for bio-spiritual claims: that mental purification through meditation, chanting mantras, prayer, and mindfulness can influence the physical, cognitive, and moral character of future children. This translation localises Turkmendag and Liaw’s (2022) argument of epigenetic responsibilisation: GS cosmologies imagine parents as responsible for the epigenetic improvement of their children through wellness. This hybridisation of epigenetics, wellness, and spirituality extends reproductive governance into the molecular interior of the body to produce a bio-spiritual therapeutic politics which suggests that wellness can shape the bodies of future citizens.

This focus on hereditariness to improve the fitness of the population resonates with older eugenic ideologies. One practitioner (Pune, 17/10/23) makes this connection explicit and calls GS ‘Ayurvedic eugenics’. GS cosmologies rework eugenic ideologies with idioms from wellness, Ayurveda, and epigenetics to suggest a therapeutic neo-eugenic logic of population improvement through mental purification. This postcolonial hybridisation suggests a uniquely Indian method of improving the population through wellness and reproduction. The specific traits that GS practitioners seek to engineer through mental purification further reveal the civilisational ambitions of GS. One practitioner (Lonavala, 18/10/23) argues that the purification of the mind improves children in terms of respect. Practitioners define respect in terms of ‘respecting the elder generation’ because ‘in Indian culture, it is first to give importance to the character, behaviour, then only education or intelligence’ (GS Practitioner, Nellore, 24/01/24). This cosmology imagines respect as an Indian-coded and hereditable biological trait which wellness can cultivate through epigenetics. GS then uses epigenetics to articulate a bio-spiritual therapeutic politics which can encode an Indian moral character and tradition into bodies of the future nation through the wellness techniques of mental purification.

## Discussing Breathing, Kinship, and Epigenetics in Wellness

Garbh Sanskar mobilises wellness as a technology of reproductive governance across interconnected registers. Practitioners imagine that pranayama and other bio-spiritual practices in wellness can cosmologically improve bodies. This understanding of national improvement through wellness conceptually extends reproduction into the realms of the sublime and civilisational. GS also uses Ayurvedic and spiritual therapies to soothe the body and mind, intertwining care with governance. These therapies help articulate a vision for reproduction which imagines a scientific nation that retains its civilisational exceptionalism. GS positions these wellness interventions as methods to improve the future child and nation through the reformation of gender, kinship, and care. Practitioners then use epigenetic imaginaries to speculate how wellness can encode these desired changes at the molecular level of future children. These logics collectively constitute a bio-spiritual therapeutic politics which intertwines wellness with reproductive governance to imagine national improvement.

GS furthers understandings of nationalist reproductive governance in three ways. First, GS demonstrates how wellness can rework older eugenic logics into new cosmologies in which prayer, meditation, and breathing create an imaginary of epigenetic improvement for individuals and the nation. Second, wellness can imbue reproductive governance with an affect that operates through idioms of gender, care, and kinship. Third, GS articulates a vision for reproduction and the nation that uses wellness to extend beyond the biological into the realms of the moral and civilisational.

This focus on localised civilisational identity does not prevent GS from braiding global resources such as international health policy and epigenetic knowledge into its offerings. Wellness can dovetail with global medical thought and local traditional medical systems to advance modern projects of nation-building through therapeutic discourses. The global circulation of these discourses helps reveal how wellness is part of other global yet localised projects of reproductive governance. The Australian National Perinatal Depression Initiative advocated for maternal mental health interventions and explained that ‘the negative impact of depression and anxiety on the developing foetus and the infant has been associated with developmental delays in the shorter and longer term’ (Highet & Purtell, 2012: 5). The US Healthy Start Program similarly provided perinatal mental health services with the motto that ‘[a] healthy start for every baby begins with a healthy mother’ (Maternal & Child Health Bureau, 2024: para. 1). The NHS is more explicit about this relationship. The NHS (2018: 6) clinical pathways argued that ‘the long-term costs from lack of timely access to good-quality perinatal mental health care is estimated to be £1.2 billion to the NHS and social services and £8.1 billion to society’. This estimate included ‘the risk of behavioural and emotional problems for the child later in life, and the likelihood of lower IQ and lower educational attainment’. Well-being interventions beyond GS position maternal well-being as a determinant of foetal and national development. Wellness is an emergent site for global reproductive governance.

## Conclusion

This article has examined how Garbh Sanskar mobilises wellness as a technology of reproductive governance in contemporary India. GS hybridises the ancient and modern with the therapeutic and spiritual to advance a syncretic project that produces new configurations of the body, mind, family, kinship, and nation. Breathwork, Ayurvedic therapies, spiritual wellness practices, and epigenetic imaginaries of mental purification are part of a bio-spiritual therapeutic politics where wellness is intimately intertwined with the governance of biological reproduction.

GS illustrates how wellness discourses travel across institutional and cultural contexts and acquire new meanings and functions through localisation, such as in postcolonial epistemologies of spirituality, morality, and civilisational improvement. Wellness allows GS to imagine the future nation using the language of well-being and reproduction. Understanding how wellness can be intertwined with reproductive governance and national aspirations can enrich critical accounts of health and power around the world.

## Data Availability

No datasets were generated or analysed during the current study.

## Annexure 1

**Table:**

Term	Definition as used in this article
Garbh Sanskar (GS)	An Indian holistic reproductive programme combining Ayurvedic and yogic traditions with contemporary wellness practices and international health policy. GS centres offer breathwork, guided meditation, counselling, and Ayurvedic therapies to expectant and hopeful couples, framing these interventions as tools for individual, familial, and national improvement.
Bio-spiritual politics	A form of governance in which the management of biological reproduction is inseparable from the management of spiritual and metaphysical self.
Psy-globalisation	Duncan’s (2018) term for the transnational circulation and localisation of therapeutic discourses. GS participates in psy-globalisation by braiding Euro-American wellness frameworks with South Asian philosophical and medical traditions.
Reproductive governance	Morgan and Roberts’s (2012) concept describing how states, social movements, and other actors regulate reproduction through legal, medical, and cultural mechanisms.
Somatic nationalism	Alter’s (1994) concept describing a utopian vision of nationalist reform that takes the body as its primary object of discipline and improvement.
Epigenetics	The biological study of changes in genetic expression without changes to DNA structure.
Ayurveda	A system of traditional Indian medicine with roots in ancient Sanskrit texts.
Pranayama	Yogic breathing practices, translating approximately as ‘control of the life force’.
Nuclearisation	Socio-economic process by which extended or joint family households give way to smaller nuclear family units.
